# UTRs and Ago-2/miR-335 Complex Restricts Amylin Translation in Insulinoma and Human Pancreatic β-Cells

**DOI:** 10.3390/ijms25179614

**Published:** 2024-09-05

**Authors:** Zhanar Kudaibergenova, Satyabrata Pany, Elizabeth Placheril, Aleksandar M. Jeremic

**Affiliations:** Department of Biological Sciences, The George Washington University, Washington, DC 20052, USA; zhannkud81@gwmail.gwu.edu (Z.K.); panysb@gmail.com (S.P.); eplacheril@gmail.com (E.P.)

**Keywords:** type-2 diabetes mellitus, human islet amyloid polypeptide, protein translation, argonaute, miRNAs, promoter, gene expression

## Abstract

Amylin promoter and transcriptional factors are well-established, inducible factors in the production of the main amyloidogenic pancreatic hormone, human islet amyloid peptide (hIAPP) or amylin. However, posttranscriptional mechanisms driving hIAPP expression in pancreas remain enigmatic, and hence were explored here. The translational assay revealed that both 5′ and 3′ untranslated regions (UTRs) of hIAPP restricted expression of the luciferase constructs only in constructs driven by the hIAPP promoter. Bioinformatics analysis revealed several putative seed sequences for a dozen micro RNAs (miRNAs) in hIAPP’s 3′ UTR. miR-182, miR-335, and miR-495 were the most downregulated miRNAs in stressed human islets exposed to endoplasmic reticulum (ER) or metabolic stressors, thapsigargin (TG) or high glucose (HG). Correspondingly, miR-335 mimics alone or in combination with miR-495 and miR-182 mimics significantly and potently (>3-fold) reduced hIAPP protein expression in HG-treated cultured human islets. siRNA-mediated silencing of Ago2 but not Ago1 significantly stimulated hIAPP expression and secretion from transfected, HG-treated human islets. Conversely, ectopic expression of Ago2 in hIAPP-expressing RIN-m5F cell line driven by CMV promoter reduced hIAPP intracellular protein levels. Collectively, the results point to a novel and synergistic role for hIAPP promoter, 5/3′ UTRs and Ago-2/miR-335 complex in post-transcriptional regulation of hIAPP gene expression in normal and metabolically active β-cells.

## 1. Introduction

The global prevalence of type 2 diabetes mellitus (T2DM) has increased dramatically in the last couple of decades making it a major global health issue. Epidemiological studies estimate approximately 462 million individuals or 6.3% of the world’s population are currently affected by T2DM [1]. Clinically, the manifestation of T2DM is seen as chronically elevated blood glucose levels or hyperglycemia due to the inability of pancreatic β-cells to adequately produce/secrete glucose-regulating hormone, insulin, and/or the insulin resistance of peripheral tissues [2]. The two pathological processes are connected as pancreatic β-cells up-regulate transcription and secretion of insulin to compensate for the decreased insulin sensitivity, which often depletes β-cell insulin stores and induce ER-stress [3].

Loss of pancreatic β-cell mass, evoked in part by aggregation and toxicity of the pancreatic β-cell hormone, human islet amyloid polypeptide (hIAPP) or amylin, is another salient feature of T2DM in humans and primates but not rodents [2,4,5,6]. Under normal physiological conditions, the pancreatic β-cells produce and co-secrete two main pancreatic hormones, hIAPP and insulin, which regulate glucose homeostasis. IAPP is co-secreted due to elevated blood glucose levels to reduce gastric emptying and create a feeling of satiation and it suppresses the release of glucagon [7]. In contrast to insulin, the *hIAPP* gene is weakly expressed in human islets under normal physiological conditions [8]. However, chronically elevated blood glucose levels seen in T2DM stimulate hIAPP production and secretion [9], which then accumulates in the pancreas as soluble oligomers and insoluble protein aggregates or amyloid plaques, causing toxicity to the pancreatic β-cells [5,7]. Above the threshold concentration, the physiological (monomeric) form of hIAPP forms a dimer which then assembles into oligomers in concentration- and time-dependent manner [10,11]. Through continuous fibrillization (polymerization) process, hIAPP oligomers form protofibrils and finally insoluble, mature, and full-grown fibrils. hIAPP-derived amyloid deposits were detected in the pancreas in over 90% of T2DM patients, as compared to ≤7% of healthy subjects [7,12], suggesting its causal role in disease progression. Thus, the dynamics of hIAPP turnover (its production and degradation) could be an important factor in hIAPP intracellular and extracellular oligomerization, aggregation, and toxicity. Despite recent progress in understanding hIAPP pathology, the molecular mechanisms driving its translation in β-cells under normal and diabetes-associated pathological conditions, such as hyperglycemia, remain to be elucidated.

IAPP is encoded by a single-copy gene on chromosome 12, and it comprises three exons [13]. The last two exons encode the full (89 aa) preproIAPP hormone [13]. Following the cleavage of its N terminal 24 aa signal sequence, IAPP is then converted into proIAPP form [5,8]. Partially processed proIAPP (and proinsulin) then undergo similar posttranslational processing by prohormone convertases PC2 and PC1/3, as well as carboxypeptidase E, to produce a 37 aa long mature form [4]. The active hIAPP monomeric form adopts soluble, predominantly random coil conformation, and is co-released together with insulin from islet β-cell granules in glucose- and nutrient-dependent manner [5,8,14]. In line with their synergistic physiological action, hIAPP and insulin promoters share common transcription factors such as PDX1 and ISL1 [15,16,17]. Interestingly, transcriptomics analysis revealed that high glucose (HG) stimulates a disproportional ~10-fold increase in IAPP’s mRNA, while HG is mildly stimulatory to insulin transcription [9]. Given these findings, it has been proposed that posttranscriptional modifications and differential promoter regulation could account for this differential expression of insulin and IAPP [18,19]. Unlike insulin, the IAPP promoter includes three FoxA2 binding sites, among which the proximal FoxA2 binding site (−606 bp) is highly conserved across species [19]. Relevantly, the major glucose-inducible gene in β-cells, a thioredoxin-interacting protein (TXNIP), stimulates IAPP transcription by increasing the expression of its main transcription factor, FoxA2 [19]. Additionally, hIAPP promoter activity requires calcium-responsive elements that are yet to be identified [14].

In addition to this well-studied hIAPP transcriptional mechanisms [5,15], emerging evidence points to important translational control of IAPP synthesis in pancreatic β-cells [20]. Untranslated regions (UTRs) of mRNA have an important and universal role in the post-transcriptional regulation of gene expression [21]. Notably, the 3′ UTR of hIAPP is unusually long (~1.5 kb), which could potentially serve as a regulatory binding site for many post-transcriptional factors and processing [22]. Although hIAPP 5′ UTR is significantly shorter than its 3′ UTR, it may also impact translation through secondary structure modifications, recruitment of regulatory RNA binding proteins, and modulation of upstream open reading frames (uORFs) and upstream AUGs (uAUGs) [23]. However, little is known about the actual contribution of the UTRs on hIAPP expression particularly under diabetes-relevant (high glucose) conditions. Like these high metabolic conditions, short-term ER stress tends to stimulate hIAPP transcription in the pancreatic human islets together with other ER-stress responsive proteins [24]. Importantly, amylin’s counterpart hormone, insulin, and its secretion from pancreatic β-cells is negatively regulated at the posttranscriptional level by components of the multiprotein RISC complex, argonaute 2 (Ago2), and micro-RNA-375 (miR-375) and miR-335 through β-cell secretome. [25,26]. It is possible that a similar Ago/miR complex also regulates hIAPP production in same cells. In contrast to insulin, the post-transcriptional mechanisms and factors involved in hIAPP synthesis remain enigmatic, and hence were investigated here. Our studies identified miRNA-responsive regulatory elements within hIAPP 3′ UTR that regulate hIAPP protein synthesis in normal and high glucose-exposed pancreatic β-cells. We also investigated and confirmed the essential role of the cell’s main translational repressor, the RISC complex, in hIAPP synthesis and release in high-glucose-stimulated human islet cells.

## 2. Results

### 2.1. hIAPP 5′ and 3′ UTR Independently Regulate Protein Translation in Rat Insulinoma Cells

**Figure 1:**
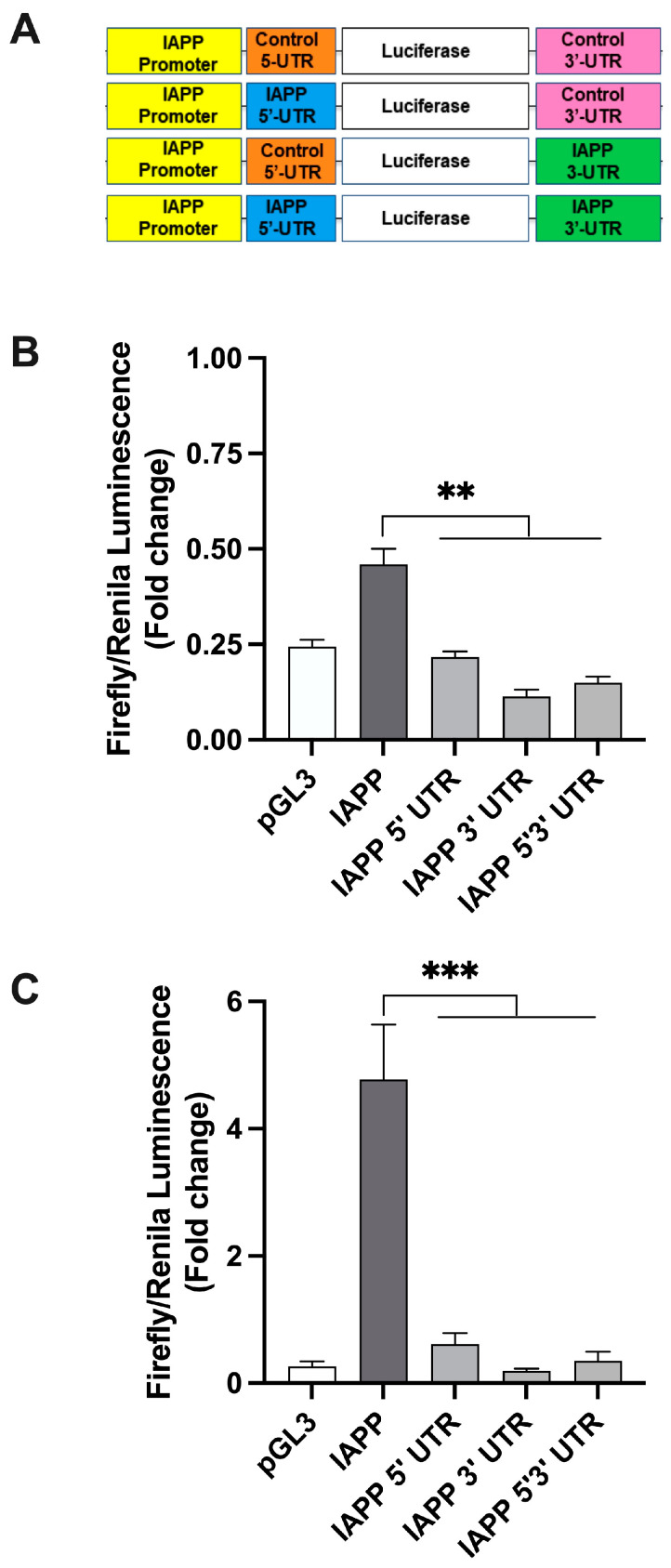
hIAPP 5′ and 3′ UTRs regulate protein translation in pancreatic cells. RIN cells were co-transfected with the designated plasmid constructs encoding firefly luciferase and the renilla vector ((**A**) for short-term (3 h, (**B**)) or longer incubation times (12 h, (**C**)). (**A**) Design map of vectors containing IAPP promoter and control or IAPP 5′/3′-UTRs. (**B**) Short term (3 h) expression analysis of IAPP-promoter-driven translation constructs. (**C**) Prolonged (12 h) expression analysis of IAPP-promoter-driven translation constructs. Normalized translational activities of IAPP-promoter-driven constructs containing hIAPP 5′ and/or 3′ UTRs were statistically compared to control IAPP construct featuring generic 5′3′ UTRs. Significance established at ** *p* < 0.01 and *** *p* < 0.001 (IAPP vs. IAPP UTRs) n = 3, ANOVA followed by Tukey post hoc comparison test.

**Figure 2:**
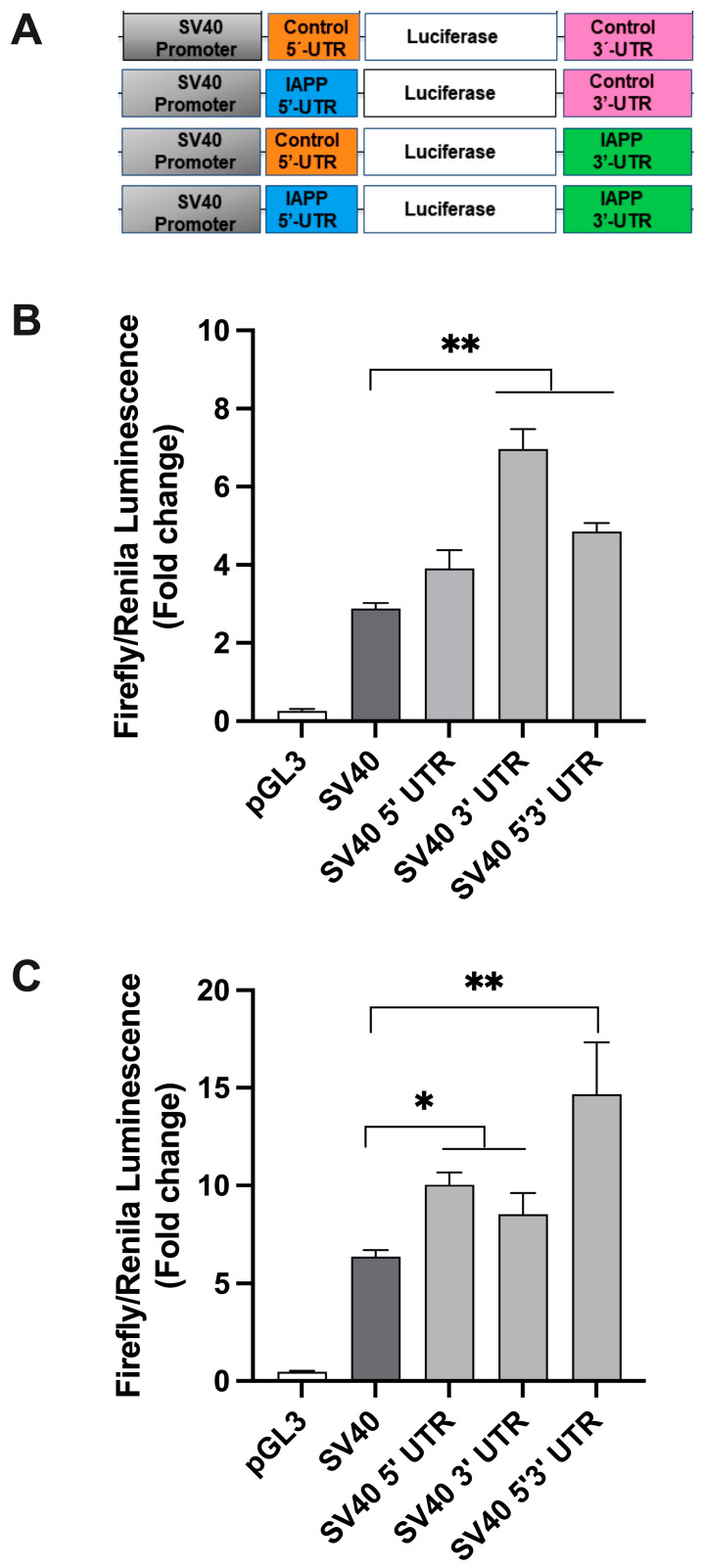
Translational activity of SV40-promoter-driven constructs in RIN cells. RIN cells were co-transfected with the designated plasmid constructs encoding firefly luciferase and the renilla vector ((**A**) for short-term (3 h, (**B**)) or longer incubation times (12 h, (**C**)). (**A**) Design map of vectors containing viral, SV40 promoter, and control or IAPP 5′/3′-UTRs. (**B**) Short term (3 h) expression analysis of SV40-promoter-driven translation constructs. (**C**) Prolonged (12 h) expression analysis of SV40-promoter-driven translation constructs. Normalized translational activities of SV40-driven constructs containing hIAPP 5′ and 3′ UTRs with respect to control vector containing generic 5′3′ UTRs (SV40) were determined and statistically compared. Significance established at * *p* < 0.05 and ** *p* < 0.01 (SV40-control vs. SV40-IAPP UTRs) n = 3, ANOVA followed by Tukey post hoc comparison test.

Given the global role of UTRs in the control of translation efficacy and the proteome diversity, we first tested the extent to which hIAPP UTRs independently function as general regulatory elements in protein and therefore hIAPP translation. For this purpose, we created a set of recombinant expression plasmids under the control of hIAPP promoter (Figure 1A–C) or SV40 promoter (Figure 2A–C) containing hIAPP or generic (control) 5′/3′ UTRs, and the levels of luciferase activity after normalization, reflecting relative protein expression levels, were measured. The first set of translational studies was done on RINm5F cells that were transfected with the IAPP promoter-driven constructs (Figure. 1A), for a short-term, 3 h (Figure 1B). The luciferase expression assay revealed that in the IAPP-promoter-driven constructs, replacement of control UTRs with hIAPP’s 5′ UTR, 3′ UTR or their combination had a significant (*p* < 0.01, n = 3) and marked (~50–70%) inhibitory effect on luciferase expression levels (Figure 1B). To analyze regulatory effects of hIAPP’s UTRs on extended timeline, the second set of translation studies were performed using a longer, 12 h transfection protocol (Figure 1C). As expected, extending the transfection time by 9 h enhanced the luciferase signal four-fold (Figure 1C). Despite this signal enhancement, we again observed a significant (*p* < 0.001, n = 3) decrease in relative luciferase levels in constructs containing IAPP translational regulatory elements (IAPP 5′ UTR, 3′ UTR and 5′3′ UTR), as compared to the controls (IAPP-promoter-driven construct containing generic 5′3′ UTRs, Figure 1C).

### 2.2. hIAPP UTRs and SV40 Promoter Do Not Restrict Protein Translation in Insulinoma β-Cells

In contrast to IAPP-promoter-driven constructs (Figure 1A–C), inclusion of IAPP 5′ and/or 3′ UTRs in SV40-driven constructs (SV40 3′ UTR, SV40 5′3′ UTR, Figure 2A) significantly (*p* < 0.01, n = 3) stimulated luciferase signal respective to a control SV40-construct (Figure 2B). This stimulatory effect of SV40 in our translational assay was also observed in cells transfected with constructs for longer periods of time (12 h, Figure 2C). Notably, the SV40 constructs showed the highest protein expression values when both amylin’s UTR regions were present (SV40 5′3′ UTR) reaching >10-fold increase respectively to control (SV40 construct, *p* < 0.01, n = 3) (Figure 2B,C). Thus, hIAPP promoter is essential for inhibitory action of hIAPP’s 5′ and 3′ UTRs on protein translation.

### 2.3. 3’UTR of hIAPP Contains Seed Sequences for Several T2DM-Relevant miRNAs

To further verify the findings of our reconstituted translation studies, we used bioinformatics tools to identify complementary miRNAs to hIAPP 3′ UTR. The RISC complex components, micro-RNAs (miRNAs) and argonaute (Ago) proteins, play a pivotal regulatory role in protein translation in many cells including pancreatic cells by interacting with regulatory elements within 5′ and 3′ UTRs of respective transcripts [27,28]. Hence, it is conceivable that one or more of pancreatic miRNAs may mediate restrictive effects of amylin’s UTRs in our translation assay (Figure 1). Sequence analysis revealed a dozen miRNAs that were partially complementary to seed-sequences in amylin’s 3′ UTR (Figure S1). Putative miR’s seeding position (underlined) was predicted using TargetScan program. Several miRNAs matched two or more seed-sequences in amylin’s 3′ UTR, most notably miR-330 (Figure S1) indicating their possible regulatory involvement in hIAPP translation. Interestingly, certain hIAPP’s putative miRNAs, such as miR-124 and miR-495, were previously linked to hIAPP transcription and diabetes pathology, respectively [19,29].

### 2.4. miR-182, miR-335, and miR-495 Levels Are Downregulated in Stressed Human Islets

**Figure 3:**
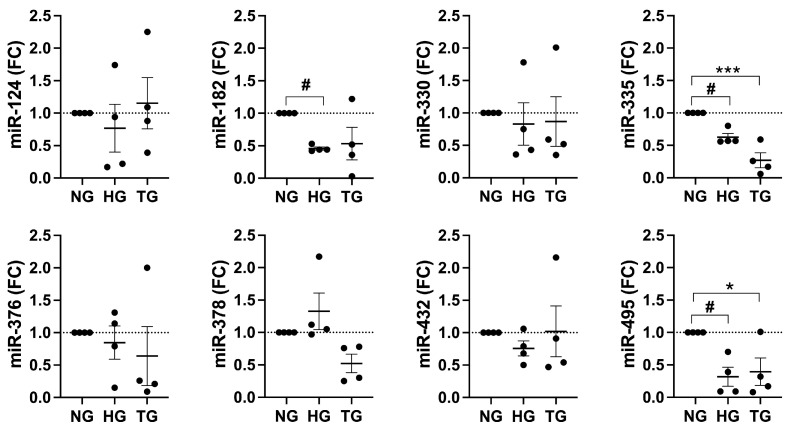
miRNAs expression analysis in HG-challenged human islets. Freshly isolated non-diabetic human islets were cultured in the presence of high (20 mM) glucose (HG) or 1 μM thapsigargin (TG) for 24 h. Relative miRNA levels were analyzed by RT-qPCR and changes in miRNA expression levels in treatments, after normalizing to a ubiquitously expressed miRNA SNORD44, were expressed as a fold change (FC) relative to control (normal glucose, NG) cells. Significance established at # *p* < 0.05, n = 3 (HG vs. NG), and * *p* < 0.05, *** *p* < 0.001, n = 3 (TG vs. NG), ANOVA followed by Tukey post hoc comparison test.

**Figure 4:**
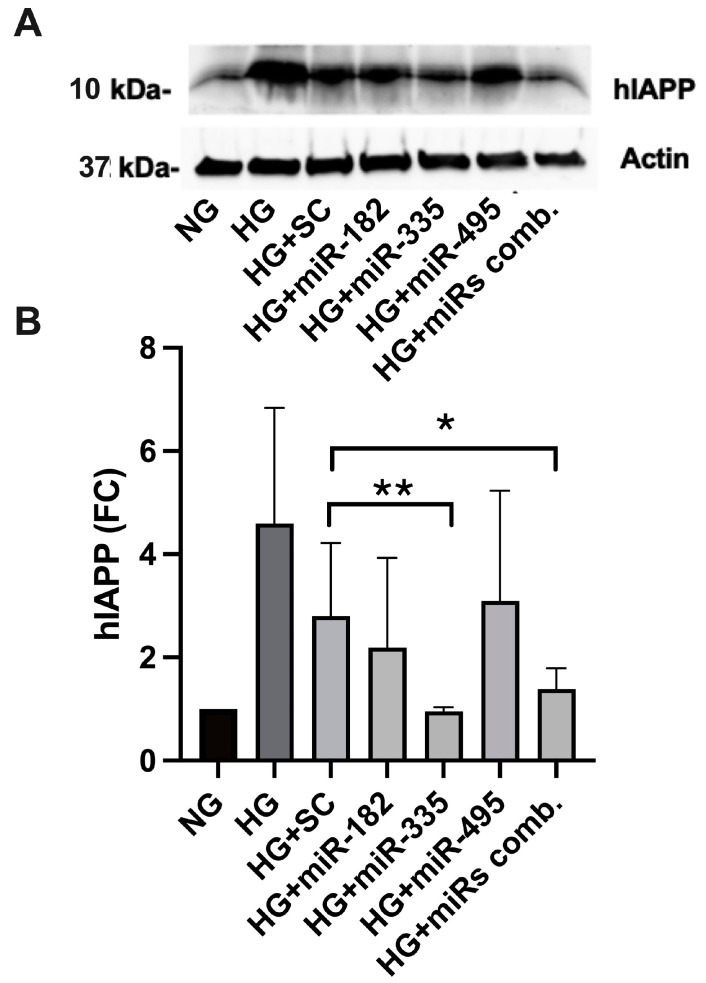
Western blot analysis of miRNA-driven hIAPP expression in human islets. (**A**) Freshly isolated non-diabetic human islets were cultured in the presence of high (20 mM) glucose (HG) or normal (5 mM) glucose (NG) supplemented with scrambled (SC) or antisense miRNAs (182, 335, 495) or their combination (miRs comb.) for 72 h, and changes in hIAPP protein expression analyzed by western blot. (**B**) hIAPP signal was normalized against beta-actin and expressed as fold change from control (NG) samples (set to 1). Significance established at ** *p* < 0.01, n = 3, (HG + SC vs. HG + miR-335) and * *p* < 0.05, n = 3, (HG + SC vs. HG + miRs combination), ANOVA followed by Tukey post hoc comparison test.

It is possible that seed sequences in hIAPP UTRs and complementary miRNAs regulate (arrest) hIAPP translation in resting and/or stressed β-cells. In line with this idea, using specific miRNA primers (Table S2) and qPCR analysis we quantified the cellular levels of several predicted hIAPP’s miRNAs. Transcripts levels of miR-182, miR-335, and miR-495, were markedly (>2-fold) and significantly downregulated in cultured human islets exposed to metabolic and ER stressors, high glucose (HG) and thapsigargin (TG), as compared to controls (Figure 3). Interestingly, for some putative amylin’s miRNAs, HG or TG had minimal inhibitory effect or were even mildly stimulatory, such as miR-378 (HG, Figure 3). These results suggest a scenario where a subset of hIAPP complementary miRNAs is downregulated during high glucose and/or ER stress conditions, thus alleviating hIAPP translational arrest. To further verify this idea, we used the mimics of these three most consistently downregulated miRNAs in our loss of function studies (Figure 4).

### 2.5. miR-Mimics Complementary to hIAPP 3′ UTR Seed Sequences Confine hIAPP Protein Expression in HG-Treated Cultured Human Islets

From a large pool of hIAPP’s putative miRNAs, qPCR analysis revealed that miR-182, miR-335 and miR-495 were consistently the most downregulated miRNAs in stressed human islets (Figure 3). Here, using the “mimic” oligonucleotides of these miRNAs, we determined their functional contribution in hIAPP synthesis in HG-challenged human islet cells. Freshly isolated human islets were transfected with the transfection complex containing each of the above miRNA mimics or their combination at a concentration of 5 nM siRNA, transfection reagent, and serum-free media for 24 h. The islets were further incubated with HG (20 mM Glc) for 48 h. Protein extracts were prepared for non-transfected (control), scrambled (SC), and mimics-transfected human islets. hIAPP protein levels in human islet extracts were then determined by western blot and densitometry. A significant (*p* < 0.01, n = 3) and marked (~4-fold) drop in hIAPP protein levels was observed in HG-treated/miR-335 transfected islets relative to the scrambled or control samples exposed to high glucose (HG, Figure 4). The level of hIAPP expression reduction (~3.7-fold) in islets containing all three mimics was comparable to miR-335 transfectants (Figure 4). hIAPP protein expression remained unchanged in miR-495 treated human islets and was slightly downregulated by ~15% with miR-182 mimics (Figure 4). Thus, western blot analysis demonstrated that the miR-335 is both necessary and sufficient to arrest hIAPP translation evoked by HG.

### 2.6. Catalytic Active Ago-2 Form Limits hIAPP Synthesis in Human and Rat Pancreatic β-Cells

**Figure 5:**
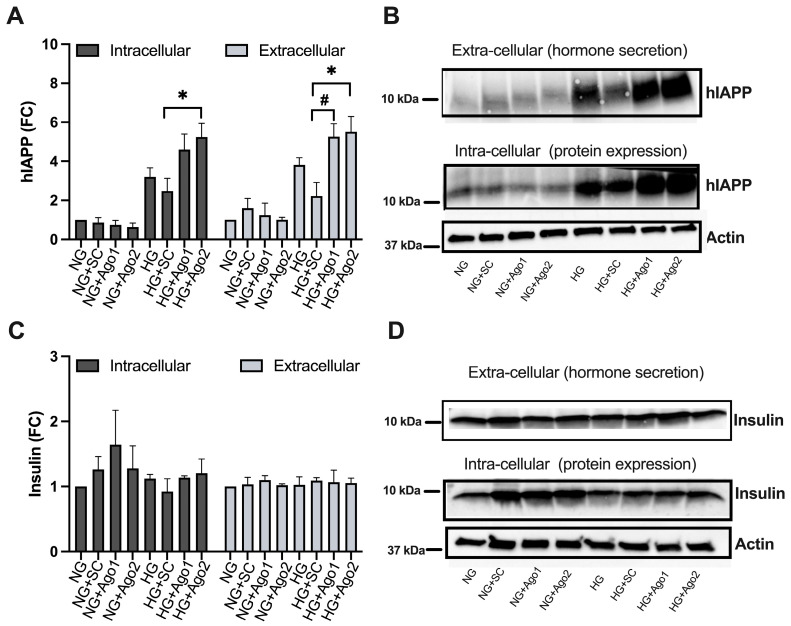
Effect of Ago-1/2 siRNAs on β-cell hormone protein expression and secretion in human islets. The human islets were cultured under normal- (NG) or high-glucose (HG) conditions supplemented with scrambled (SC) or Ago1/2 siRNAs for 72 h, and changes in human amylin (hIAPP) and insulin protein expression were analyzed by western blot. (**A**) Intracellular and extracellular hIAPP signals were normalized against beta-actin and expressed as fold change from control normal glucose (NG) samples (set to 1). (**B**) Representative western blots of intracellular and extracellular hIAPP contents under various conditions. (**C**) Intracellular and extracellular insulin signals were normalized against beta-actin and expressed as fold change from control (NG) samples (set to 1). (**D**) Representative western blots of intracellular and extracellular insulin protein contents under various conditions. Significance established at * *p* < 0.05, n = 3 (HG + Ago2 vs. HG + SC), and # *p* < 0.05, n = 3 (HG + Ago1 vs. HG + SC), ANOVA followed by Tukey post hoc comparison test.

Argonaute proteins are critical components of the RISC complex involved in the regulation of gene expression. In humans, four argonaute proteins (Ago1–4) were described, and Ago2 is the only argonaut family member that possesses endo-nucleolytic capability. Functionally, the difference between Ago1 and Ago2 is not clear. To gain mechanistic insights into RISC’s complex role in hIAPP translation, we functionally repressed Ago1 and Ago2 proteins using synthetic siRNAs (Figure 5). For this experiment, we transfected human islets with siAgo1, siAgo2 or scrambled (siSc) oligos (10 nM) to inhibit Ago1 and Ago2 expression/activity under normal and high glucose (HG) conditions. After 72 h of transfection and 24 h HG treatment, intracellular (cell lysates) and extracellular (secretory) protein contents were analyzed by western blot. In agreement with the findings from the miRNA overexpression experiment (Figure 4), western blot analysis revealed a significant (*p* < 0.05, n = 3, Figure 5A,B) ~2.2-fold increase in mature (processed) hIAPP intracellular and extracellular protein levels in Ago2-transfected human islets as compared to control (SC-transfected, HG-treated, HG + SC) islets. Phenocopying this stimulatory effect of Ago2 siRNA on amylin expression and secretion from human islets, Ago1 siRNA transfection stimulated a significant release of hIAPP from these cells reflected by ~2.4-fold increase in hIAPP extracellular levels (*p* < 0.05, n = 3, Figure 5A,B). Transfection of islets with Ago1 construct raised hIAPP intracellular protein (expression) levels in HG-treated islets (HG + Ago1) ~1.6-fold relative to control cells (HG + SC), however, not reaching the significance (*p* = 0.1, n = 3, Figure 5A). This Ago2- and Ago1-mediated upward trend in hIAPP expression and secretion was not observed in Ago1/2-transfected human islets cultured under normal (5 mM Glc, NG) glucose concentrations or in HG-treated human islets transfected with random (scrambled, SC) sequences (Figure 5A,B) indicating Ago-2 and to a lesser extent Ago1 involvement in translational repression of hIAPP in metabolically active (glucose stimulated) human islet cells. Interestingly, siRNA Ago1 and Ago2 did not have any significant effect on) expression or release of fully and/or partially processed (pro-) insulin under either resting or stimulated conditions (*p* > 0.05, n = 3, Figure 5C,D). Western blot analysis also revealed that high glucose, although stimulatory for hIAPP (Figure 5A,B) and for some of its putative miRNAs (Figure 3 and Figure S1), did not significantly alter intracellular (protein expression) levels of Ago1 or Ago2 (Jeremic and Kudaibergenova unpublished observation).

**Figure 6:**
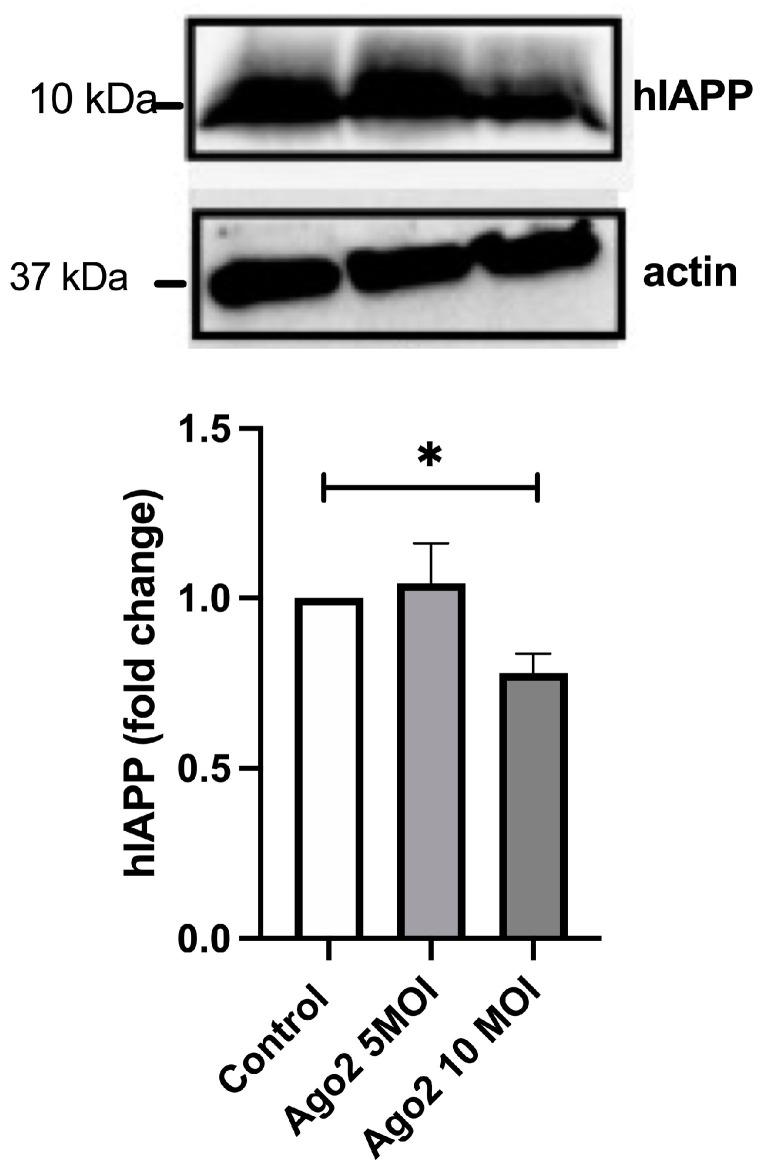
Ago-2 controls hIAPP expression in RINm5F cells. Changes in protein levels of hIAPP in lentivirus-transfected cells encoding Ago2 was analyzed by western blot. Cells were transfected at 5 and 10 MOIs. hIAPP signal was normalized to a housekeeping gene (beta-actin) and expressed as fold change from control RIN cells. Significance established at * *p* < 0.05, n = 3, ANOVA followed by Tukey post hoc comparison test.

The specific effect of siAgo2 on hIAPP but not on insulin turnover in human islets ruled out the broad, non-specific effect of our treatments on general secretion and points out to translational control of hIAPP production and release from these cells by Ago2. This posttranscriptional control of hIAPP turnover was further verified in Ago2-transfected RIN-m5F cells overexpressing hIAPP under the strong, cytomegalovirus (CMV)-driven promoter. We detected moderate (~25%) but significant decrease in hIAPP intracellular protein levels following transfection and expression of Ago2 lentiviral construct at a higher virus titer (10 MOI) (*p* < 0.05, n = 3, Figure 6). Collectively, functional studies (Figure 4, Figure 5 and Figure 6) together with translational and bioinformatic studies (Figure 1, Figure 2, Figure 3 and Figure S1) support the important role for the RISC complex and 5′/3′ UTRs in repressing translation of hIAPP transcripts in primary cells and cells overexpressing it ectopically under normal and metabolically relevant (high-glucose) conditions.

## 3. Discussion

Recent studies point to an important role of miRNAs and Ago proteins in insulin secretion and regulation of gene expression in pancreatic β-cells and link them to T2DM progression [19,25,26,28,29]. However, the role of these two important post-transcriptional regulatory elements in hIAPP gene expression is yet to be determined. In the current study, we found that the 5′ and particularly 3′ UTRs from the hIAPP transcript significantly and markedly restricted the expression of the luciferase gene in insulinoma β-cells. However, this restrictive action of the hIAPP 5′ and 3′ UTRs on protein translation is only detected when constructs’ expression was driven by the native (hIAPP) promoter. We postulate that the inhibitory effects of the hIAPP 5′ and 3′ UTRs on gene expression is conveyed, at least in part, at the posttranscriptional level as this regulatory effect was not recapitulated with the constructs driven by SV40 promoter. Like regulation of human aggrecan gene [30], replacement of a native inducible (IAPP) promoter with viral, constitutively active (SV40) promoter converted the luciferase gene expression from inhibitory to stimulatory. In the case of the aggrecan gene, the differential effects of its untranslated regions on the two types of promoters used in the study, the native and CMV, were attributed to the presence or absence of TATA region, respectively [30]. The presence of TATA regulatory elements in hIAPP promoter may allow for similar (inhibitory) action of hIAPP UTRs on its gene transcription.

Modulatory effects of the hIAPP 5′ and 3′ UTRs on luciferase protein expression suggested their canonical translational role in hIAPP expression. Indeed, the post-transcriptional control of hIAPP expression by its UTR’s was demonstrated in Ago2-transfected RIN-m5F cells constitutively overexpressing hIAPP under the CMV promoter, which showed reduced hIAPP production compared to control cells. In agreement with the findings in insulinoma cells, the current study suggests the involvement of Ago-2/miR-335 RISC complex in restricting hIAPP protein expression and secretion in human islets under elevated metabolic flux evoked by high glucose. Interestingly, Ago-2/miR-335/miR-375 RISC complex was found to restrict insulin release from human and murine pancreatic β-cell lines [25,26]. Consistent with these findings, from ~12 putative miRNAs that are complementary to seed sequences in amylin’s 3′ UTR, only mir-335 and, to a lesser extent, miR-182 levels were downregulated in HG-challenged islet cells, and their mimics were effective in reducing hIAPP protein levels. Although miR-495 intracellular levels were also strongly downregulated by HG treatment, its mimics remained inefficient in modulating hIAPP protein levels, revealing a relatively narrow functional pool of miRNAs acting in hIAPP expression. In contrast to islet miRNA levels, HG did not alter mRNA or protein levels or Ago1/2. Importantly, silencing of catalytically active Ago2 and to lesser extent Ago1 form, had significant impact on hIAPP expression and secretion in HG-exposed human islet β-cells. These findings suggest that high glucose stimulates hIAPP translation and secretion primarily by controlling (reducing) intra-islet miRNA-335/182 levels, which in turn limit Ago-2 catalytic activity and/or action (but not its expression) on hIAPP transcripts, thereby preventing hIAPP mRNA degradation and/or translational repression by the RISC complex. In addition to this direct effect on hIAPP translation, the Ago2/miR-335 complex may block its secretion from β-cells indirectly by perturbing the β-cell secretome apparatus and reducing expression and function of exocytotic SNARE proteins, which negatively impact insulin release from these cells [25,26].

However, it is possible that other Ago isoforms or other miRNAs not tested in our study may also control hIAPP mRNA and protein turnover in the pancreas. Therefore, future studies are required to confirm the full repertoire of RISC complex constituents that participate in regulation of hIAPP translation under various metabolic conditions. Collectively, combination of cis-regulatory elements within hIAPP promoter and its 5′ and particularly 3′ UTR allows for a tight (post)transcriptional control of hIAPP gene expression under normal and high glucose conditions associated with T2DM. Future studies are needed to determine the impact of these miRNAs on the stability (turnover) of hIAPP transcripts and its implications for hIAPP toxicity and T2DM.

## 4. Materials and Methods

### 4.1. Cell Culture 

Control and hIAPP-stably expressing rat insulinoma (RIN-m5f) β-cells were used in our transfection and translational studies, per protocols described previously [24]. RIN-m5f cells (ATCC, Gaithersburg, MD) were propagated in RPMI 1640 medium (ATCC), supplemented with 10% (*v*/*v*) fetal bovine serum and 1% penicillin/streptomycin) and split bi-weekly. Insulinoma cells (passage no. 15–40) were seeded 96-micro well plates at 50,000–60,000 cells per well.

For ex vivo studies, non-diabetic human islets with >90% purity and viability were used. Islets were obtained through the Integrated Islet Distribution Program (IIDP). Fresh human islets were separated from non-islet material under a dissecting microscope, yielding a purity of ≥95% (based on dithizone staining); 30–40 intact or partially dissociated islets were suspended in CMRL media (ATCC) containing 1% (*v*/*v*) fetal bovine serum albumin and 1% penicillin/streptomycin and plated on 48-well non-adherent cell culture plastic plates. Partially mechanically dissociated islets were used for transfection studies. The islets were cultured at 37 °C in a humidified incubator with 5% CO_2_ for indicated periods of time. To induce ER or metabolic stress, cell cultures were treated with 1 μM thapsigargin (0–8 h) or high glucose (20 mM) for 24 h, respectively.

### 4.2. Transfections

miRNA and Ago-2 siRNA transfections: Mission transfection reagent (Sigma-Aldrich, St. Louis, MO, USA) and Lipofectamine 3000 (Invitrogen, Waltham, MA, USA) were used for the transfections of miRNA mimics and siRNAs, respectively. Transfection complexes were prepared by preparing 5 nM miRNA mimics (Sigma-Aldrich) or 10 nM siRNAs (Santa Cruz Biotechnology, Dallas, TX, USA) in appropriate transfection reagent and serum-free islet growth medium, followed by 15 min of incubation to allow formation of transfection complex. Transfection complex was then gently poured over the cultured islets and incubated with human islets in antibiotic-free media at 37 °C for 24 h. Following transfection, media was replaced with normal growth islet media and human islets incubated for an additional 48 h prior to addition of high glucose media.

Lentiviral transfection: Lentivirus-mediated Ago-2 overexpression were carried out in hIAPP-overexpressing RIN-m5f cells [24]. Ready in use lentivirus particles were purchased from Origene Technologies (Rockville, MD, USA). After 24 h of recovery time, lentivirus particles encoding Ago-2 were added at the MOI (multiplicity of infection) of 5 and 10 and incubated for 24 h. Polybrene (8 mg/mL) was used during the transduction procedure to enhance transfection efficiency or viral integration. Lentivirus-containing medium was replaced with normal growth medium, and the insulinoma cells were allowed to grow for an additional 48 h at 37 °C. After 72 h of post transduction, proteins were collected for western blot analysis.

### 4.3. Translation Activity Assay

hIAPP’s 5′-UTR (152bp) and 3′-UTR (1554 bp) (Figure S2) were cloned (Mutagenex Inc. Suwanee, GA, USA) into SV40-driven pGL3 reporter vector encoding firefly luciferase (Promega, Madison, WI, USA). 5′-UTR and 3′-UTR sequences of hIAPP mRNA in human islet were characterized by 5′ and 3′-RACE (Rapid Amplification of cDNA Ends) (Thermo-Fisher Scientific, Waltham, MA, USA) and confirmed with available reference sequence (NP_000406.1) The 5′-UTR of IAPP was inserted into HindIII and NcoI sites downstream of luciferase gene and the 3′-UTR of IAPP was inserted into XbaI and BamHI sites upstream of luciferase gene. The hIAPP promoter was localized to 2000 bp upstream of transcription start site in IAPP gene (NC-018923.2) and then isolated from purified genomic DNA of human islets. SV40 promoter was replaced with IAPP promoter using restriction sites, BaglII and HindIII. The inserts and their proper orientations were confirmed by DNA sequencing.

In total, ~5 × 10^4^ RIN-m5F cells were plated on 96-well tissue culture plates using RPMI 1640 media (Thermo Fisher Scientific, Waltham, MA, USA) supplemented with 10% (*v*/*v*) fetal bovine serum (Thermo Fisher Scientific, Waltham, MA, USA). At 24 h following plating, RIN-m5F cells were co-transfected for 3 or 12 h with thymidine kinase Renilla promoter vector (Promega, Madison, WI, USA) and the hIAPP or SV40 5′/3′ UTR constructs using the FuGENE HD (Promega, Madison, WI, USA) transfection reagent. Transfected cells were then treated with vehicle (DMSO) or 1 μM of thapsigargin (TG) for an additional 8 h. Relative firefly and renilla luciferase activities were detected using the Dual-Glo luciferase assay system (Promega, Madison, WI, USA) following the manufacturer’s instructions. Firefly luminescence was normalized with Renilla luminescence for each sample/well to account for differential transfection efficiency. The luciferase activity, reflecting translational efficiency of the constructs, is reported as mean ± SEM of normalized signal (fold change, FC).

### 4.4. Western Blot Analysis

The western blot method was used to quantify cellular and extracellular IAPP and insulin protein levels along with the ER stress marker, BiP. RIN-m5F or human islet cells were incubated with thapsigargin or high glucose for 8 and 24 h hours, respectively, in the presence or absence of translational inhibitors. Following treatments, the cells were lysed in IPA buffer and protein extracts separated on 4–15% tris/glycine SDS/PAGE. The resolved proteins were transferred to nitrocellulose membranes and nonspecific IgG-binding sites were blocked by incubation with 5% nonfat dry milk in the wash buffer (PBS) for 2 h. After blocking and washing, the blot was cut into strips and probed with a rabbit BiP primary antibody (Abcam, Cambridge, UK) (1:1000) or rabbit insulin primary antibody (Abcam, 1:2000) and HRP-conjugated goat anti-rabbit secondary antibody (Abcam, 1:3000). hIAPP and actin expression levels were quantified with HRP-conjugated anti-hIAPP (Santa Cruz, 1:500) and anti-actin (Santa Cruz, 1:100) monoclonal mouse antibodies. hIAPP and insulin primary antibodies are immune-reactive against nascent and processed (mature) peptide forms. The band intensity, reflecting relative protein expression, were photographed and quantified using the gel doc imaging system from Bio-Rad Laboratories (Hercules, CA, USA) and its associated software (ImageLab, version 6.1).

### 4.5. Quantitative Real-Time PCR

Total RNA was extracted from RIN-m5F cells using TRIzol reagent (Life Technologies, Carlsbad, CA, USA) and the Direct-zol RNA Miniprep kit (Zymo Research, Irvine, CA, USA) according to the manufacturer’s instructions. Total RNA was further purified from contaminating DNA using in-column DNA digestion with RNase-free DNase, following the manufacturer’s instructions. cDNA was synthesized from 250 ng of RNA using multiScribe reverse transcriptase and random primers (Applied Biosystem, Waltham, MA, USA) or MystiCq miRNA cDNA synthesis kit (Sigma-Aldrich) according to the manufacturers’ protocol. RT-qPCR was performed using a specific Bio-Rad CFX 96 real-time PCR system. Transcript levels were quantified from the reverse-transcribed cDNA using specific mRNA primers (Table S1) or miRNA primers (Table S2), and iTaq Universal SYBR Green supermix using the following thermal cycling protocol: polymerase activation 95 °C, 2 min; DNA denaturation 95 °C, 5 s; annealing and extension 60 °C, 30 s; repeat 40 cycles. mRNA and miRNA in samples were normalized to actin and SNORD44, ubiquitously expressed small nucleolar miRNA, respectively and their relative expression levels calculated using the 2^−ΔCt^ method [24,31].

### 4.6. Statistical Analysis

The Graph Pad Prism program version 9 (GraphPad, La Jolla, CA, USA) was used for plotting and statistical analysis of collected data. The data are presented as mean ± SEM from at least three independent sets of experiments. The unpaired Student’s *t*-test or one-way ANOVA followed by the Tukey post hoc test was used for pairwise comparisons among groups when appropriate. Significance was established at *p* < 0.05.

## 5. Conclusions

The findings of this study, derived from rat insulinoma and human islet cells, are consistent with a novel and synergistic action of the hIAPP promoter and its UTRs in post-transcriptional regulation of the hIAPP gene expression in pancreatic β-cells. Bioinformatics and functional studies point to the 3′ UTR-nested Ago-2/miR-335 complex as an important inhibitor of hIAPP translation and synthesis in metabolically active pancreatic β-cells.

## Data Availability

The raw data supporting the conclusions of this article will be made available by the authors on request.

## Supplementary Materials

The following supporting information can be downloaded at: https://www.mdpi.com/article/10.3390/ijms25179614/s1.

## Abbreviations

ANOVA: analysis of variance; BIP, binding immunoglobulin protein; BRE, brefeldin-A; ER, endoplasmic reticulum; hIAPP, human islet amyloid polypeptide; IRES, internal ribosome entry site; RT-qPCR, real-time quantitative PCR; SV40, simian virus 40; TG, thapsighargin; TU, tunicamycin; 7-methylguanylate (m7G); uORF, upstream open reading frame; UPR, unfolded protein response; UTR, untranslated region.
