# Supplementary material for: UTRs and Ago-2/miR-335 Complex Restricts Amylin Translation in Insulinoma and Human Pancreatic β-Cells
Source: Int J Mol Sci. 2024 Sep 5;25(17):9614. doi: 10.3390/ijms25179614 (PMC11394793; doi:10.3390/ijms25179614)
Supplement: Supplementary file 1 [file ijms-25-09614-s001.zip › ijms-3160507-supplementary.pdf]

Supporting Information

UTRs and Ago-2 / miR-335 complex restricts amylin translation in insulinoma and human pancreatic β-cells

Zhanar Kudaibergenova[1], Satyabrata Pany[1], Elizabeth Placheril[1] and Aleksandar M. Jeremic[1]

[1]Department of Biological Sciences, The George Washington University, Washington, DC 20052, USA

Supplemental materials contain the following information: *Figure S1*: Bioinformatics analysis of miRNAs seed-sequences in hIAPP 3'UTR, Figure *S2:* Control and hIAPP UTRs cloning sequences, *Tables S1, S2*: mRNA and miRNA primer sequences used for gene expression analysis.

1. **has-miR-124**
5' ...UCCCUUUAUGAAGUGUGCCUUAG... (UTR) **Position 1(1528-1535)**
|||||||
3' CGUAAGUGGCGCACGGAAUU (miR)

2. **has-miR-143**
5' ...UUAACAGUGCCCUUUUCAUCUCC... (UTR) **Position 1(193-199)**
||||||| |||||||
3' CUCGAUGUCACGA----AGUAGAGU (miR)

5' ...AGCCGAGAUUGCACCACUGCACU... (UTR) **Position 2(731-737)**
||||| |||||||
3' UGGUCUCUACGUCG-UGACGUGG (miR)

3. **has-miR-155**
5' ...GGUAGCUUUGGAAGUAGCAUUAA... (UTR) **Position 1(1369-1376)**
|||||||
3' UGGGGAUAGUGCUAAUCGUAAUU

4. **has-miR-182**
5' ...CCAGCAACAACUUUGGUGCCAUU... (UTR) **Position 1(22-28)**
|||||||
3' UCACUUAAGAUGGUCACGGUAU (miR)

5. **has-miR-330 – overlapped sequences in 3' UTR (highlighted in grey)**
5' ...UUAAAAGUAUAAAACUGCUUUGU... (UTR) **Position 1 (997-1003)**
|||||||
3' AGAGACGUCCGGCACACGAAACG (miR)

5' ...UUAAAAGUAUAAAACUGCUUUGU... (UTR) **Position 2 (1298-1304)**
|||||||
3' AGAGACGUCCGGCACACGAAACG (miR)

5' ...GAAGAUGAAAUGCUUUGCUUUGA... (UTR) **Position 3 (1303-1310)**
|||||||
3' AGAGACGUCCGGCACACGAAACG (miR)

6. **has-miR-335**
5' ...GGACCAGGCGCGGUGGCUCUUGC... (UTR) **Position 1 (505-511)**
|||||||
3' UGUAAAAAGCAAUAACGAGAACU (miR)

7. **has-miR-376**
5' ...AAAAAUAAGCAUUUCAAUCUAAG... (UTR)**Position 1 (1467-1471)**
||||||
3' AUGAGUAUCUUCCUCUUAGAUG (miR)

5' ...AUAUUUAUAAUAAAUUCUAUGUC... (UTR)**Position 2 (1625-1631)**
||| |||||||
3' UGCACCUUAAAGGAGAUACAA (miR)

8. **has-miR-378**

5' ...CAGGCAGAUCACAAGGUCAGGAG... (UTR) **Position 1 (558-564)**
|||||||
3' UGUGUCCUGGACCUCAGUCCUC (miR)

9. **has-miR-495**

5' ...GUCUGUGUGUCUGAUGUUUGUUG... (UTR) **Position 1(230-236)**
|||||||
3' UUCUUCACGUGGUACAAACAAA (miR)

5' ...UUUUUAAAGUCUAUUGUUUGUUG...(UTR) **Position 2 (950-956)**
|||||||
3' UUCUUCACGUGGUACAAACAAA (miR)

10. **has-miR-506**

5' ...UCCCUUUAUGAAGUGUGCCUUAG... (UTR) **Position 1(1529-1535)**
|||| |||||||
3' AGAUGAGUCUUCCCACGGAAUG

11. **has-miR-634**

5' ...AUUGUUUGUUGUGCUUGCUGGUA... (UTR) **Position 1 (962-969)**
|||| |||||||
3' CAGGUUUCAACCCC-ACGACCAA (miR)

12. **has-miR-1185 – overlapped sequences in 3' UTR (highlighted in grey)**

5' ...UCUGGAAACUCUUUG-CUGUAUAA... (UTR) **Position 1(1189-1196)**
||| |||||||
3' UACUCUCAGAGGGGGACAUAUA

13. **has-miR-4789 – overlapped sequences in 3' UTR (highlighted in grey)**

5' ...CUGGAAACUCUUUGCUGUAUAAG... (UTR) **Position 1 (1190-1196)**
||||||
3' GUAUGUGUAUAGUCCACAUAUG (miRNA)

5' ...UGGUAGCGGUAGUGAGUGUAUAG... (UTR) **Position 2 (1590-1596)**
|||||||
3' GUAUGUGUAUAGUCCACAUAUG (miRNA)

Figure S1. Bioinformatics analysis of IAPP mRNA 3'-UTR. Databases comprising predicted and validated miRNA-target interactions were used. miRanda (http://www.microrna.org/microrna/home.do), DIANA-microT-CDS, mirDB (http://ophid.utoronto.ca/mirDIP) and TargetScan (http://www.targetscan. org/vert_71) collect predicted miRNA targets. Two databases, miRtarbase (http://mirtarbase.mbc.nctu.edu.tw) and DIANATarbase (http://diana. imis.athena-innovation.gr/DianaTools/index.php?r1/4site/index), contained validated miRNA targets. Putative miR's seeding positions (underlined) were predicted using TargetScan (http://www.targetscan. org/vert_71).

Control 5'-UTR[1]: 5'- (HindIII) AAGCTT GGCATTCCGGTACTGTTGGTAAAGCCA CCATGG (NcoI)

Control 5'-UTR[2]: 5'- (SpeI)
ACTAGTTAGCAAATGAGGGGGTAAATATTCCAGTGGATACAAGCTTGGACTCTTTTCTTGAAGCTTT
CTTTCTATCAGAAGCATTTGCTGATATTGCTGACATTGAAACATTAAAAG CCATGG (NcoI)

Control 3'-UTR: 5'- (XbaI) TCTAGA GTCGGGG CGGCCGGCC (FseI)

hIAPP 5'-UTR (152 bp):
5'GGGTATATAAGAGCTGGATTACTAGTTAGCAAATGAGGGGGTAAATATTCCAGTGGATACAAGCTT
GGACTCTTTTCTTGAAGCTTTCTTTCTATCAGAAGCATTTGCTGATATTGCTGACATTGAAACATTAAA
AGAAAATTTGAGAAGCA

IAPP 3'UTR (1554 bp):
5'AGGACAATGTAACTCTATAGTTATTGTTTTATGTTCTAGTGATTTCCTGTATAATTTAACAGTGCCCT
TTTCATCTCCAGTGTGAATATATGGTCTGTGTGTCTGATGTTTGTTGCTAGGACATATACCTTCTCAA
AAGATTGTTTTATATGTAGTACTAACTAAGGTCCCATAATAAAAAGATAGTATCTTTTAAAATGAAATG
TTTTTGCTATAGATTTGTATTTTAAAACATAAGAACGTCATTTTGGGACCTATATCTCAGTGGCACAG
GTTTAAGAACGAAGGAGAAAAAGGTAGTTTGAACCTTGGTAAATTGTAAACAGCTAATAATGAAGTTA
TTCTTGACATGAGAAAATCAGTAATTGGACCAGGCGCGGTGGCTCTTGCCTGTAATCCCAGCACTTT
GGGAGGCCGAGGCAGGCAGATCACAAGGTCAGGAGTTCGAGACCAGCCTGACCAACATGGTGAAA
CCCTGTCTCTACTAAAAATACAAAAATTAGCCGGGGGTGGTGACATGTGCCTGTAATCCCAGCTACT
CAGGAGGCTAAGGCAGGAGAATCGCTTAAACCCAGGAGGCGGAGGTTGCAGTGAGCCGAGATTGC
ACCACTGCACTCCAGCCTGGGTGGCAGAGTGAGACTCGTCTCAAAAAAAAGAAAGAAAATTAGTAAT
TGTAAGTACCCCTGATAAGCAAATTAGTAATTGTCAATACCCCTGTTAAGCAATTCCTTTTTGCAGTA
TATTTCTGAAATGACAGAATGCTGTTTTAAAAACAAAGAAATAAAATCCTGCTCCTGACTCGGTCAAA
ATATTTTTTAAAGTCTATTGTTTGTTGTGCTTGCTGGTACTAAGAGGCTATTTAAAAGTATAAAACTGC
TTTGTATCCATGAGGGTTTCATTGTGTGTTAGCAGCAGTGAGCTTCTATTAAATGTATATGTCATTTAT
TTTGTTTAAGTGGCTTTCAGCAAACCTCAGTCATATTCTTATGCAGGGTATTGCGAAACAACTTGTGT
TCTATTAATCGTGTCTTCAATTAAAAGACCACAGACTTCTGGAAACTCTTTGCTGTATAAGAATTATTT
CTTTTGTTTAACAAATTAGACATTTCTGGCAGAGGTTATGTATATGATACACTTTTTTTGATAGCAGCT
GCAATGTTGGACAGAAGATGAAATGCTTTGCTTTGAGTCAGATTCTTATGAATATCTGCTTTTCCCTG
ACTTTGAGTTAGGTAGCTTTGGAAGTAGCATTAATTCAGATAAACTGCCATCATGCTGCGTTATGCCA
TTTCTAAAGACACTCAACTTGTACTTTTAAAAAAATAGAAAAAATAAGCATTTCAATCTAAGTGGAAAT
TTGACTCATTGACTTACATTTCTAAGTTAAAATTTCCCTTTATGAAGTGTGCCTTAGGTTACCAAATTG
TAGAGGCTTTCGTTGGTGGTGGTAAGTGGTAGCGGTAGTGAGTGTATAGAGGCAGGGAAATATATT
TATAATAAATTCTATGTCATGAATTACATATTGAAATAAATAGGTGAATATACAAATTTATA

Figure S2. Control and hIAPP UTRs cloning sequences.

Primers	Sequences
hIAPP	F: 5'-AGCTACACCCATTGAAAGTCATC-3'<br>R: 5'-GATGAGAGAATGGCACCAAAGT-3’
Insulin	F: 5'- TGTCCTTCTGCCATGGCCCT-3'<br>R: 5'-TTCACAAAGGCTGCGGCTGG-3'
β-actin	F:5'-AGGCACCAGGGCGTGAT-3'<br>R:5'-GCCCACATAGGAATCCTTCTGAC-3'
TXNIP	F: 5'-ACAGAAAAGGATTCTGTGAAGGTGAT-3'<br>R: 5'-GCCATTGGCAAGGTAAGTGTG-3'
BIP	F: 5’-GAACGTCTGATTGGCGATGC-3’<br>R: 5’-ACCACCTTGAACGGCAAGAA-3’

Table S1. mRNA primer sequences used for gene expression.

**Primers**	**Sequences (3’ to 5’)**	**Accession No.**	**Position**
hsa-miR- 124-3p	UAAGGCACGCGGUGAAUG CCAA	MIMAT0000422	53–74
hsa-miR-155-3p	CUCCUACAUAUUAGCAUUAACA	MIMAT0004658	43-64
hsa-miR- 182-3p	UGGUUCUAGACUUGCCAA CUA	MIMAT0000260	67-87
hsa-miR-376A-3p	AUCAUAGAGGAAAAUCCACGU	MIMAT0000729	44-64
hsa-miR-330-3p	GCAAAGCACACGGCCUGCAGA GA	MIMAT0000751	57-79
hsa-miR-335-3p	UUUUUCAUUAUUGCUCCUGAC C	MIMAT0004703	52-73
hsa-miR-378A-3p	ACUGGACUUGGAGUCAGAAGG C	MI0000732	43-64
hsa-miR- 432-3p	CUGGAUGGCUCCUCCAUGU CU	MIMAT0002815	62-82
hsa-miR-495-3p	AAACAAACAUGGUGCACUUCUU	MIMAT0002817	50-71
hsa-miR-506-3p	UAAGGCACCCUUCUGAGUAGA	MIMAT0002878	71-91

Table S2. miRNA primer sequences used for gene expression.
